# Hypoxia alleviates dexamethasone-induced inhibition of angiogenesis in cocultures of HUVECs and rBMSCs via HIF-1α

**DOI:** 10.1186/s13287-020-01853-x

**Published:** 2020-08-06

**Authors:** Miaomiao Chai, Ce Gu, Qihua Shen, Jiaxing Liu, Yi Zhou, Ziyang Jin, Wanli Xiong, Yan Zhou, Wensong Tan

**Affiliations:** State Key Laboratory of Bioreactor Engineering, School of Bioengineering, East China University of Science and Technology, 130 Meilong Rd, Shanghai, 200237 People’s Republic of China

**Keywords:** Mesenchymal stem cell, Human umbilical vein endothelial cell, Angiogenesis, Dexamethasone, Hypoxia

## Abstract

**Background and aim:**

Inadequate vascularization is a challenge in bone tissue engineering because internal cells are prone to necrosis due to a lack of nutrient supply. Rat bone marrow-derived mesenchymal stem cells (rBMSCs) and human umbilical vein endothelial cells (HUVECs) were cocultured to construct prevascularized bone tissue in osteogenic induction medium (OIM) in vitro. The angiogenic capacity of HUVECs was limited in the coculture system. In this study, the effects of the components in the medium on HUVEC angiogenesis were analyzed.

**Methods:**

The coculture system was established in OIM. Alizarin red staining and alkaline phosphatase staining were used to assess the osteogenic ability of MSCs. A Matrigel tube assay was used to assess the angiogenic ability of HUVECs in vitro. The proliferation of HUVECs was evaluated by cell counting and CCK-8 assays, and migration was evaluated by the streaked plate assay. The expression levels of angiogenesis-associated genes and proteins in HUVECs were measured by qRT-PCR and Western blotting, respectively.

**Results:**

Dexamethasone in the OIM suppressed the proliferation and migration of HUVECs, inhibiting the formation of capillary-like structures. Our research showed that dexamethasone stimulated HUVECs to secrete tissue inhibitor of metalloproteinase (TIMP-3), which competed with vascular endothelial growth factor (VEGF-A) to bind to vascular endothelial growth factor receptor 2 (VEGFR2, KDR). This effect was related to inhibiting the phosphorylation of ERK and AKT, which are two downstream targets of KDR. However, under hypoxia, the enhanced expression of hypoxia-inducible factor-1α (HIF-1α) decreased the expression of TIMP-3 and promoted the phosphorylation of KDR, improving HUVEC angiogenesis in the coculture system.

**Conclusion:**

Coculture of hypoxia-preconditioned HUVECs and MSCs showed robust angiogenesis and osteogenesis in OIM, which has important implications for prevascularization in bone tissue engineering in the future.

## Introduction

During the past two decades, bone tissue engineering techniques have offered promising alternative approaches for the treatment of critical bone defects [1]. Cell-based constructs have received much attention in combining bone repair with stem cells. However, cell-based constructs with insufficient vascular networks may be prone to necrosis due to a lack of nutrients, oxygen, and cytokines [1–3] and would delay the bone repair process. In general, prevascularization strategies in vivo and in vitro are two main methods of tissue engineering construction [4]. Prevascularization in vitro is mainly based on the coculture of human umbilical vein endothelial cells (HUVECs) with mesenchymal stem cells (MSCs). The formation of a capillary-like network has been observed in the coculture system with growth medium [5–7]. However, our previous research found that HUVECs promoted the osteogenesis of MSCs, while the angiogenesis of HUVECs was inhibited in OIM [8].

The osteogenic environment is associated with differentiated MSCs, HUVECs, and OIM. Studies have shown that many factors have a negative effect on the angiogenesis of HUVECs, including differentiated MSCs and OIM [8–10]. However, it is still unknown which components in OIM inhibit the angiogenesis of HUVECs. OIM consists of Dulbecco’s modified Eagle’s medium (DMEM), β-glycerophosphate disodium (β-gpd), L-Vitamin C (L-Vc), and dexamethasone (DEXA). Dexamethasone is necessary in OIM and plays an indispensable role in osteogenesis. Dexamethasone promotes the expression of type I collagen, alkaline phosphatase, osteocalcin, and bone sialoprotein [11]. However, dexamethasone has been found to inhibit the formation of blood vessels in the tumor microenvironment [12] and in embryological development [13]. Dexamethasone inhibits the protein expression and activity of eNOS, which is not conducive to angiogenesis and fetal development [14]. Furthermore, dexamethasone has been proven to inhibit angiogenesis by microRNA-34a, leading to femoral head necrosis [15]. In addition, dexamethasone inhibits angiogenesis by downregulating VEGF secretion to block tumor progression [16]. However, how dexamethasone functions in the angiogenesis of HUVECs in the presence of OIM in a coculture system of MSCs and HUVECs still needs to be explored.

Vascular endothelial growth factor (VEGF) is the most important growth factor during angiogenesis. VEGF binds to vascular endothelial growth factor receptor 2 (VEGFR2, KDR), resulting in KDR phosphorylation. Phosphorylated KDR activates the downstream targets ERK and AKT, promoting the proliferation, migration, and vascular network generation of HUVECs. Tissue inhibitor of metalloproteinase (TIMP-3) is unique, endogenous protein that competes with VEGF for KDR binding in vivo [17]. TIMP-3 downregulates the phosphorylation of KDR and its downstream signaling pathways, inhibiting the angiogenesis of HUVECs. Recent studies have shown that dexamethasone can stimulate TIMP-3 expression in murine fibroblasts [18]. After administration of dexamethasone in rats, the gene expression level of TIMP-3 in the lungs was doubled compared with that in the dexamethasone-free group [19]. Furthermore, numerous studies have shown that hypoxia can downregulate TIMP-3 expression in retinal epithelial cells [20].

Hypoxia in vivo is beneficial for angiogenesis, which promotes bone repair [21]. On the one hand, hypoxia can modulate glycolysis metabolism (glucose transporter 1/3, hexokinase 1/3, lactate dehydrogenase, and pyruvate kinase M) to mediate postimplantation cell survival before the formation of blood vessels [22]. On the other hand, hypoxia-inducible factor (HIF-1α), which is expressed in hypoxic conditions, can promote blood vessel formation, which affects the transport of oxygen and nutrients to internal cells, by increasing the expression of a large number of proangiogenic factors. Therefore, hypoxia seems to be an effective approach to improve angiogenesis in bone tissue constructors in vitro.

To improve the angiogenesis of HUVECs in the presence of OIM in the coculture system, the role of dexamethasone in the inhibition of angiogenesis in the OIM coculture system still needs to be explored. Cobalt chloride (CoCl_2_)-stimulated hypoxia was used to alleviate dexamethasone-induced inhibition of HUVEC angiogenesis. The results reveal the behavior and internal mechanism of HUVECs in OIM under a coculture system and provide a strategy for prevascularization in bone tissue engineering in the future.

## Materials and methods

### Cell isolation and cell culture

All procedures on rats were performed in full compliance with the guidelines of the Ethics Committee at East China University of Science and Technology. Four-week-old Sprague Dawley rats (Shanghai SLAC Laboratory Animal Co., Ltd), which were SPF grade, male and weighed ~ 80–120 g, were used. BMSCs were isolated using the bone marrow adherence method. Cells were cultured in α minimum essential medium (α-MEM, Gibco, USA) supplemented with 10% fetal bovine serum (FBS, HyClone, USA) at 37 °C in a 5% CO_2_ humidified atmosphere. The medium was changed every 2 days. Cells at passages 3 to 5 were used in the experiments.

Primary HUVECs were purchased from ScienCell and cultured in endothelial cell growth medium (ECM, ScienCell, USA) containing 5% FBS and supplemented with 100× endothelial cell growth supplement (ECGS, ScienCell, USA). The medium was changed every 2 days. Cells at passages 3 to 8 were used in the experiments.

### Osteogenic differentiation assay

MSCs were seeded in 24-well plates at a density of 1 × 10^4^ cells/well in growth medium. After 24 h, the medium was changed to OIM that comprised DMEM (Gibco, USA) supplemented with 10% FBS, 10^−7^ M dexamethasone (Sigma, USA), 10 mM β-glycerophosphate disodium (Sigma, USA), and 50 μg/ml L-Vc (Sigma). The medium was replaced every 3 days. After induction, mineralized nodules were detected by alizarin red S staining (ARS, Sigma). Alkaline phosphatase (ALP) enzymatic activity was analyzed using an NBT/BCIP alkaline phosphatase color development kit (Beyotime, China).

For coculture of MSCs and HUVECs, MSCs were seeded in 24-well plates at a density of 1 × 10^4^ cells/well, while HUVECs were seeded at a density of 2 × 10^4^ cells/well. The initial medium was a 1:1 (v/v) mixture of α-MEM and ECM supplemented with 10% FBS and 100× ECGS. After 24 h, the medium was changed to OIM supplemented with 100× ECGS.

### In vitro tube formation assay

HUVEC monoculture or coculture with MSCs was performed on growth factor-depleted Matrigel (BD, USA) in 96-well plates with 50 μL of Matrigel/well. The capillary-like structure was viewed 6 h later. Microscopic fields containing the tube structure that formed on the gel were photographed using fluorescence inverted phase contrast microscopy. Five fields per test condition were examined. The tube length was calculated using ImageJ software.

### In vitro migration assay

HUVECs were seeded in 24-well plates at a density of 1 × 10^4^ cells/well. After the cells reached confluence, serum deprivation was performed for 16 h. A straight line was drawn in the bottom well with a sterile pipette tip (200 μL yellow tip). PBS was used to wash away the floating cells, and conditioned medium was added. After incubation for 6 h, the cell migration ability was assessed by photographing the healing of the scratches using fluorescence inverted phase contrast microscopy.

### Cell viability assay

HUVECs were seeded in 24-well plates at a density of 1 × 10^4^ cells/well with 10^−7^ M dexamethasone in ECM supplemented with 5% FBS and 100× ECGS (EGM) or OIM. The medium was replaced every 2 days. The cells were incubated at 37 °C in a 5% CO_2_ humidified atmosphere, digested with trypsin, and counted every day.

To determine the influence of hypoxia on HUVEC proliferation, CoCl_2_ (Aladdin, China) was used as a mimic for hypoxia. Cells were seeded in 96-well plates at a density of 0.5 × 10^4^ cells/well in EGM containing dexamethasone with various concentrations of CoCl_2_ (0, 50, 100, 150, 200, or 250 mM). After 24 h or 48 h, the cells were washed with PBS, and 200 μL of α-MEM comprising 10% (v/v) cell counting kit-8 (CCK-8, DOJINDO, Japan) solution was added to each well, and then the plate was incubated at 37 °C for 2 h. The absorbance at 450 nm of each well was measured by an ELx800 ELISA microplate reader (BioTek).

### Cell cycle analysis

HUVECs were cultured with or without dexamethasone (10^−7^ M) for 72 h and centrifuged at 1000 rpm, and then the cell pellet was collected and washed three times with cold PBS at 4 °C. Then, BMSCs were fixed in 70% cold ethanol in PBS. After 24 h, the cell cycle was measured by a cell cycle and apoptosis analysis kit (Beyotime). Cells were analyzed on a flow cytometer (FACSCalibur, BD).

### RNA isolation and qRT-PCR

Total RNA was extracted using TRIzol reagent (Invitrogen, USA) according to the manufacturer’s instructions. cDNA was obtained using MLV reverse transcriptase (Promega, USA). Quantitative real-time PCR (qRT-PCR) was performed as previously reported [23]. GAPDH was used as an endogenous control. All primers (Table 1) were obtained from Sangon Biotech Co., Ltd. The data were analyzed according to the ΔΔCT method.

**Table 1:** Primer sequences for qRT-PCR

Gene	Forward (5′-3′)	Reverse (5′-3′)
VEGF	TTCAAGCCATCCTGTGTGCC	CACCAACGTACACGCTCCAG
TIMP-3	CAAGGGGCTGAACTATCGGT	TCAGGGGTCTGTGGCATTGA
VEGFR2	CAGCTCACAGTCCTAGAGCG	CTGCGGATAGTGAGGTTCCG
HIF-1α	AGGTCTAGGAAACTCAAAACCTGA	CAGAAGTTTCCTCACACGCA
GAPDH	AATTCCATGGCACCGTCAAG	TGGTTCACACCCATGACGAA

### Western blotting

HUVECs were harvested and lysed in RIPA lysis buffer containing 10 mM protease inhibitors (phenylmethylsulfonyl fluoride, PMSF, Beyotime, China). A BCA protein assay kit (Beyotime) was used to determine the protein concentrations. Then, loading buffer (Beyotime) was added to the samples. The cellular proteins were separated by 10% SDS-polyacrylamide gel electrophoresis, and the separated proteins were electrically transferred onto PVDF membranes (Millipore, USA). Then, the membranes were blocked with 5% nonfat dry milk in TBST (20 mM Tris-HCl pH 7.4, 150 mM NaCl, and 0.05% Tween-20) for 1 h. The membranes were washed with TBST and probed with primary antibodies overnight at 4 °C. The bound primary antibodies (Table 2) were detected with secondary antibodies conjugated with horseradish peroxidase (HRP, Abcam, UK) and visualized by enhanced chemiluminescence (Millipore). β-Actin was used as an internal reference.

**Table 2:** Antibody used in the western blot analysis

Antibody/marker	Dilusion	Source	Code number
TIMP-3	1:1000	CST	#5673
VEGF-A	1:1000	Abcam	Ab69479
p-KDR	1:1000	CST	#2478
KDR	1:1000	CST	#2479
p-AKT	1:1000	CST	#4060
AKT	1:1000	CST	#4691
p-ERK	1:1000	CST	#4377
ERK	1:1000	CST	#4695
HIF-1α	1:1000	CST	#36169
β-Actin	1:1000	CST	#4970

### Cell transfection assay

siRNAs targeting the TIMP-3 (5′-CCAAACACUACGCCUGCAUTT-3′, 5′-AUG-CAGGCGUAGUGUUUGGTT-3′) and HIF-1α genes (5′-GCUGGAGACACAA-UCAUAUTT-3′, 5′-AUGCAGGCGUAGUGUUUGGTT-3′) (RiboBio, Guangzhou, China) were used to specifically silence TIMP-3 and HIF-1α in HUVECs, and nonspecific sequence siRNAs were used as a negative control (NC). Cells were transfected with Lipo8000 according to the manufacturer’s protocol (Beyotime). After 24 h of siRNA transfection, the knockdown efficiency was confirmed by qRT-PCR analysis.

### Statistical analysis

All experiments were repeated at least three times. All data are presented as the means ± standard deviations. Statistical differences were evaluated using Student’s two-sided *t* tests. Differences were considered statistically significant if *p* < 0.05.

## Results

### HUVECs promoted MSC osteogenesis in a coculture system with OIM

**Fig. 1:**
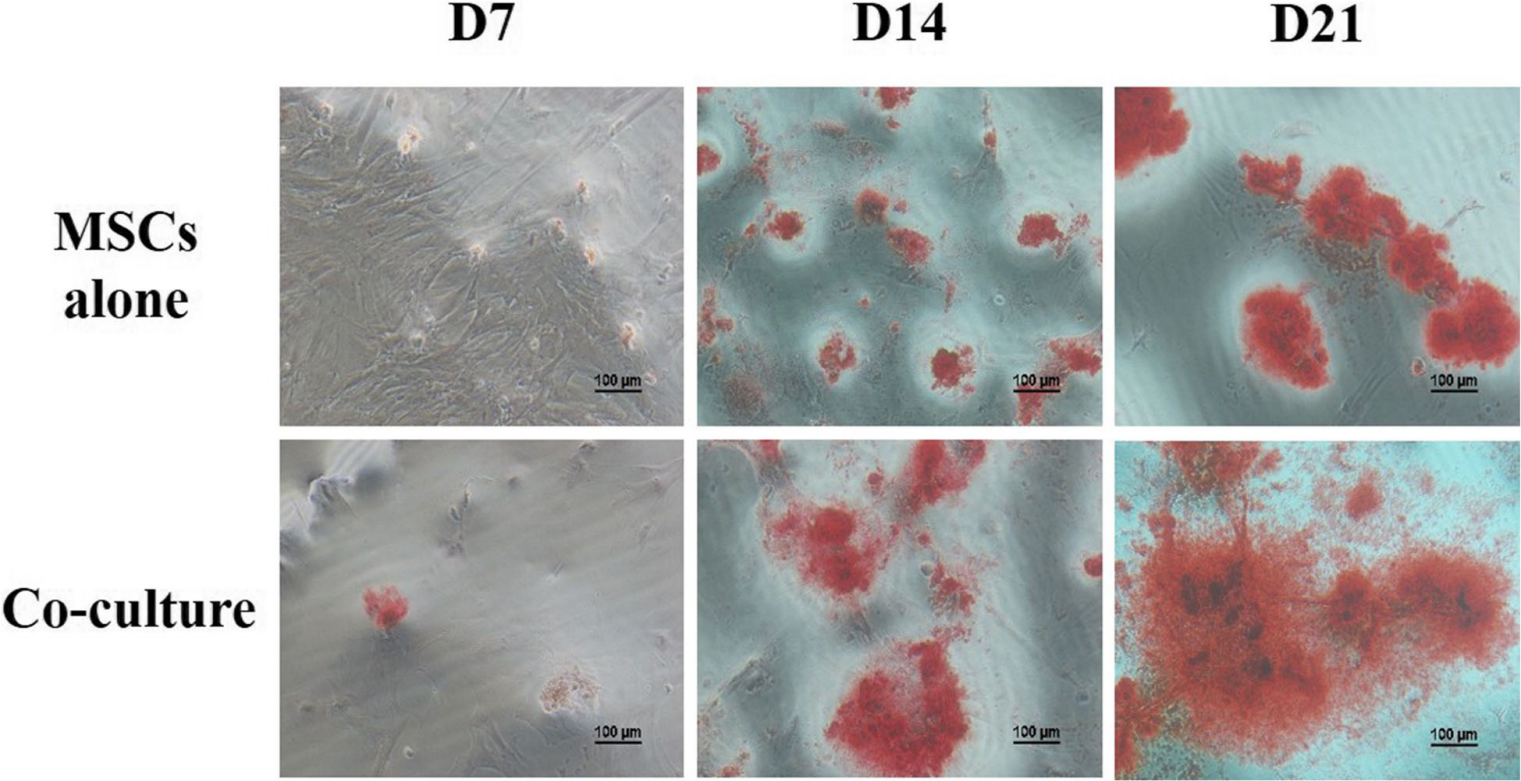
HUVECs promoted MSC osteogenesis in a coculture system with OIM. MSCs were cultured in a single culture system (MSCs alone group) or a coculture system with HUVECs (coculture group). The OIM was changed every 3 days. Alizarin red S was used to detect calcium deposition on days 7, 14, and 21. Scale bar, 100 μm

To evaluate the effect of HUVECs on the osteogenic differentiation of MSCs, MSCs were cultured with or without HUVECs in OIM for 21 days. Alizarin red S staining was performed to characterize MSC osteogenesis. More mineralized nodules appeared in the coculture system on days 7, 14, and 21 than in the monoculture system (Fig. 1). These results suggest that HUVECs promote MSC osteogenesis in OIM.

### OIM inhibited HUVEC angiogenesis

**Fig. 2:**
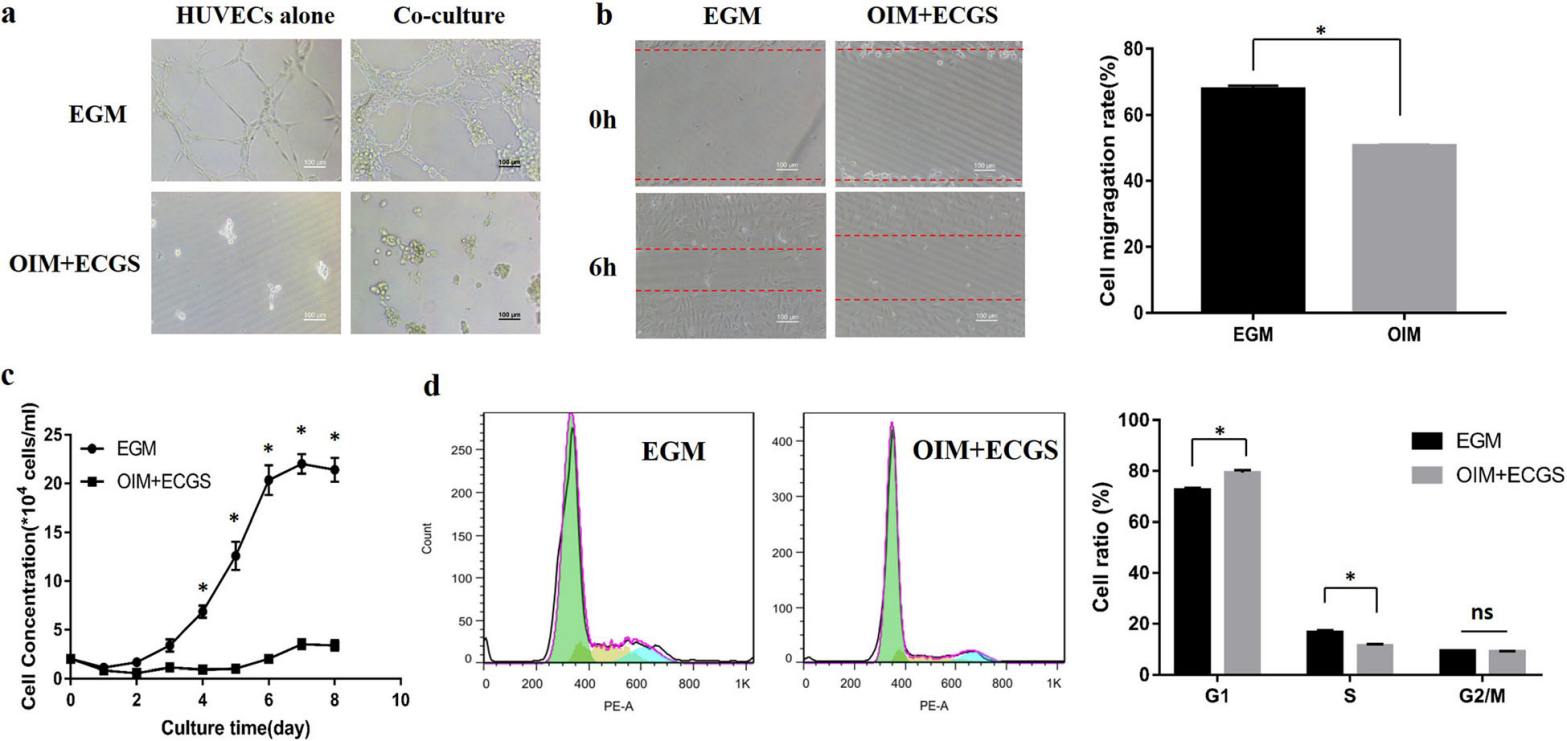
OIM inhibited HUVEC angiogenesis. **a** Angiogenesis experiment on Matrigel. Monocultured HUVECs (HUVECs alone group) or cocultured HUVECs and MSCs (coculture group) in EGM and OIM. After 6 h, photos were taken under a fluorescent inverted phase contrast microscope. **b** The streaked plate assay was used to test the migration ability of HUVECs in OIM or EGM. **c** Growth curve of HUVECs in OIM and EGM. **d** HUVECs were cultured in OIM or EGM, and the cell cycle distribution of HUVECs was detected by flow cytometry after 48 h. *p < 0.05, compared with HUVECs in EGM. *n* = 3

To observe HUVEC angiogenesis in the coculture system, a Matrigel tube formation assay was used. HUVECs alone or cocultured HUVECs and MSCs were seeded on Matrigel in EGM or OIM supplemented with ECGS and incubated for 6 h. With or without MSCs, HUVECs failed to form capillary-like structures in OIM (Fig. 2a). The migration and proliferation capacities of HUVECs were significantly suppressed in OIM compared with EGM (Fig. 2b, c). To determine how OIM impaired cell proliferation, flow cytometry was used to examine the cell cycle phases of HUVECs. After treatment with OIM, the percentage of HUVECs in the G1 phase increased, while the percentage of cells in the S phase decreased compared with that of EGM (Fig. 2d).

### Dexamethasone inhibited HUVEC angiogenesis

**Fig. 3:**
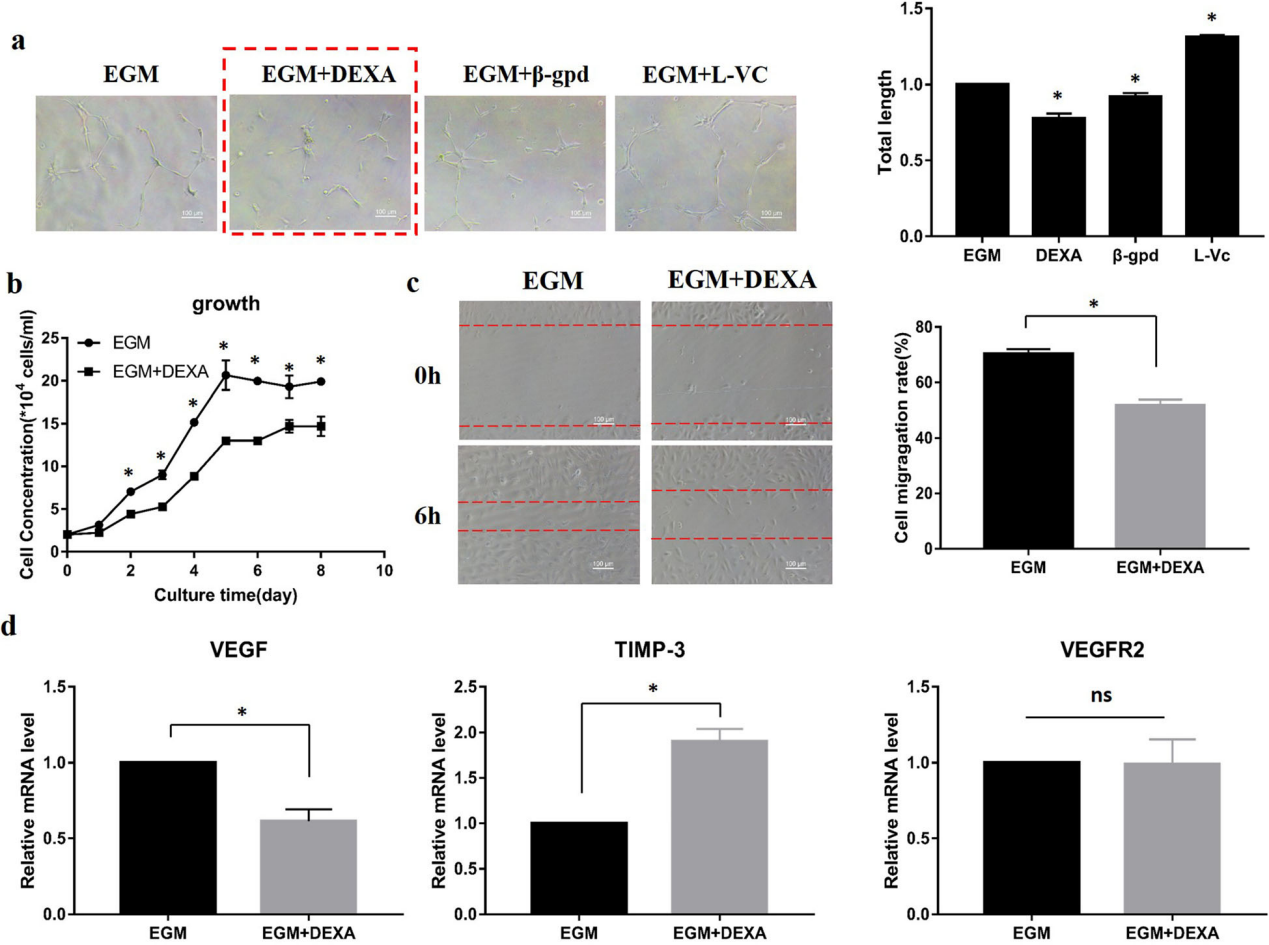
Dexamethasone inhibited HUVEC angiogenesis. **a** Dexamethasone (DEXA) was found to inhibit HUVEC angiogenesis in the Matrigel tube formation assay. Photos were taken after 6 h, and the total length of the blood vessels was quantified by ImageJ. **b** Growth curve of HUVECs in EGM treated with or without dexamethasone. **c** The streaked plate assay was used to test the migration ability of HUVECs in EGM with or without dexamethasone. **d** qRT-PCR was used to detect the expression of angiogenesis-related genes in HUVECs treated with or without dexamethasone at 72 h. *p < 0.05, compared with HUVECs in EGM. ns: *p* > 0.05, there was no significant difference. *n* = 3

To determine which component in OIM hampered HUVEC angiogenesis, the osteogenic components in OIM, dexamethasone, β-gpd, and L-vitamin C, were individually added to EGM. In the Matrigel tube assay, dexamethasone significantly inhibited the formation of capillary-like structures by HUVECs (Fig. 3a). Moreover, dexamethasone negatively affected the proliferation and migration of HUVECs (Fig. 3b, c). Subsequently, the expression of angiogenesis-related genes in HUVECs treated with or without dexamethasone was measured by qRT-PCR. The level of *VEGF*, a proangiogenic gene, was significantly decreased in the dexamethasone group (Fig. 3d). The level of *TIMP-3*, an antiangiogenic gene, was dramatically increased. There was no obvious difference in the level of *KDR*.

### Dexamethasone suppressed HUVEC angiogenesis by upregulating the expression of TIMP-3

**Fig. 4:**
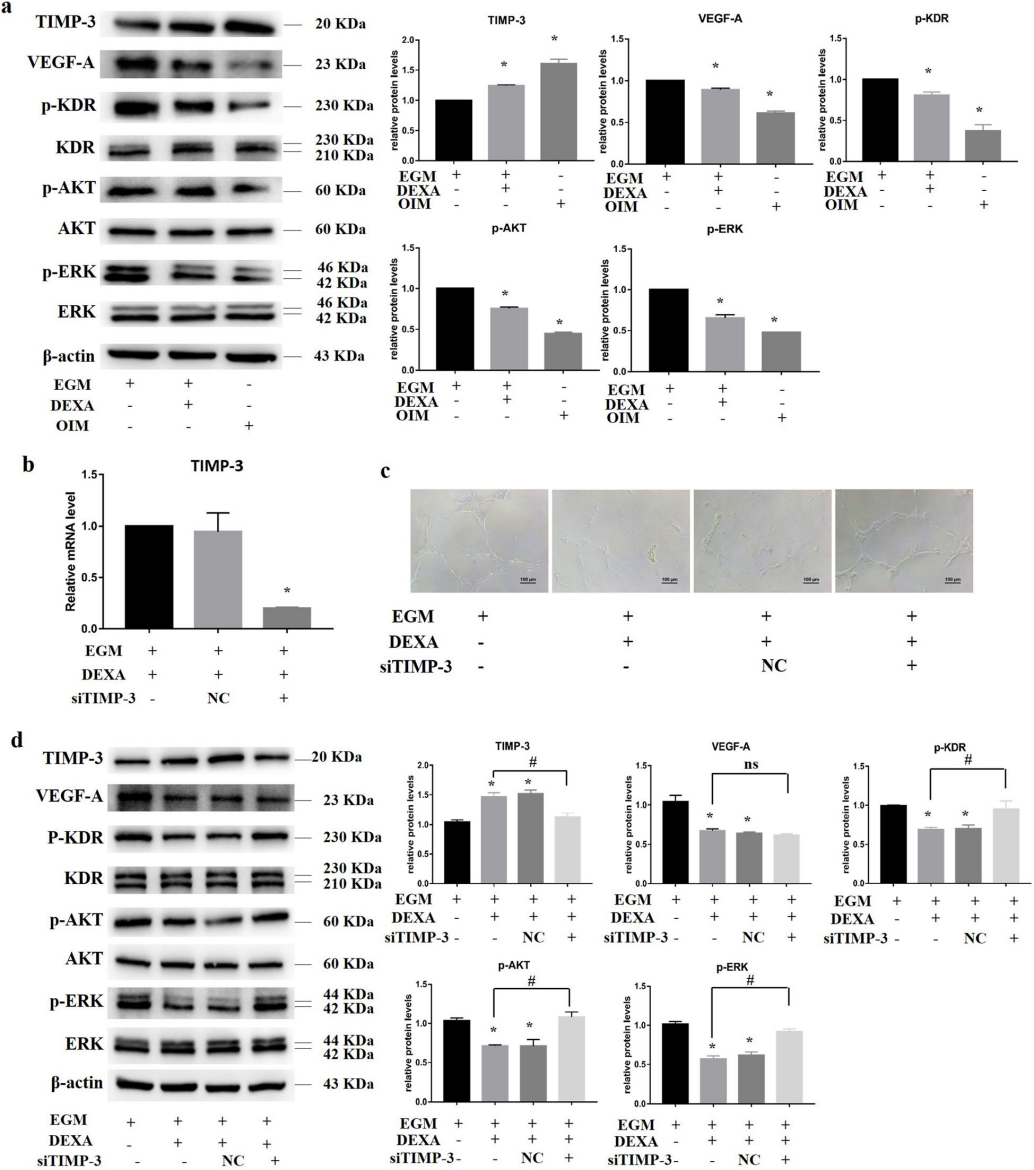
Dexamethasone suppressed HUVEC angiogenesis by upregulating the expression of TIMP-3. **a** Western blotting was performed to measure the expression of angiogenesis-related proteins at 72 h, and protein expression was quantified by ImageJ software. **b** TIMP-3 gene expression was effectively silenced by specific siRNA sequences. **c** A Matrigel tube assay was used to detect HUVEC angiogenesis after silencing TIMP-3 expression. **d** After TIMP-3 in HUVECs was silenced, Western blotting was performed to measure angiogenesis-related protein expression at 72 h. *p < 0.05, compared with HUVECs treated with the DEXA group. ^#^*p* < 0.05, compared with HUVECs treated with siNC. ns: *p* > 0.05, there was no significant difference. *n* = 3

TIMP-3 is known to block KDR signaling pathways, inhibiting HUVEC angiogenesis. To confirm the gene expression results of TIMP-3, Western blotting was performed to measure the expression of TIMP-3 and angiogenesis-related proteins. The results indicated that dexamethasone significantly upregulated the protein expression of TIMP-3, while TIMP-3 was further increased in the presence of OIM (Fig. 4a). The expression of VEGF-A, which was decreased in dexamethasone-treated HUVECs, was lowest in OIM. Similarly, the level of phosphorylated KDR, which was decreased in dexamethasone-treated HUVECs, was lowest in OIM. Furthermore, the levels of phosphorylated AKT and ERK, which are KDR downstream targets and are related to the proliferation and migration of HUVECs, were also significantly downregulated.

To further validate that TIMP-3 is an important mediator of dexamethasone-induced inhibition of angiogenesis, TIMP-3 was knocked down using a specific siRNA (Fig. 4b). Silencing TIMP-3 significantly rescued the capillary-like structures of HUVECs treated with dexamethasone (Fig. 4c). The Western blot results showed that siTIMP-3 inhibited the protein expression of TIMP-3, while the expression of VEGF-A was unchanged (Fig. 4d). In addition, the phosphorylation levels of KDR, AKT, and ERK were restored. These observations suggest that dexamethasone inhibits HUVEC angiogenesis by upregulating TIMP-3 expression.

### Hypoxia rescued the angiogenesis of HUVECs inhibited by dexamethasone

**Fig. 5:**
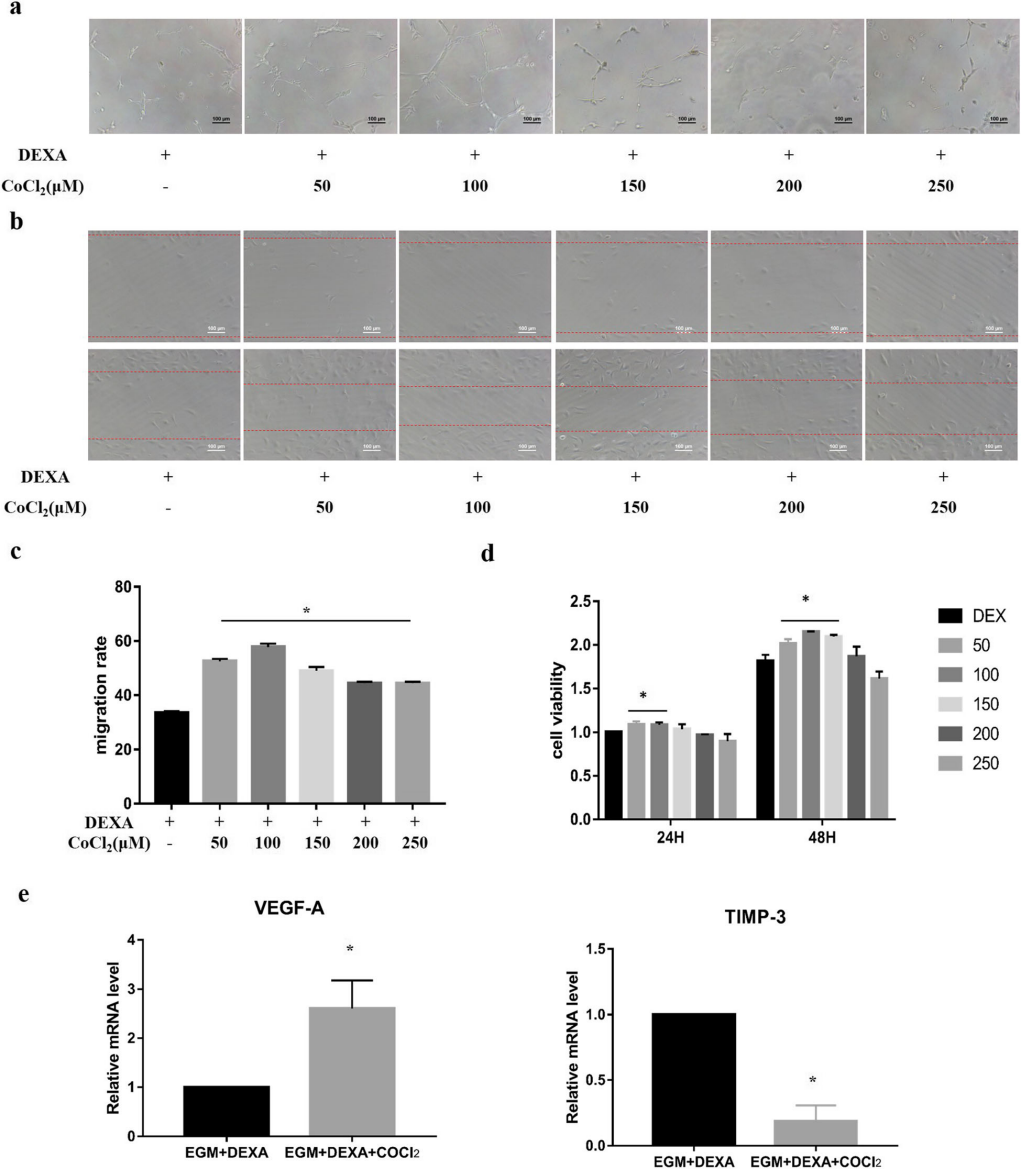
Hypoxia rescued HUVEC angiogenesis that was inhibited by dexamethasone. **a** CoCl_2_ was used to simulate hypoxia. The Matrigel tube assay was used to test the angiogenic ability of HUVECs treated with CoCl_2_ at 50, 100, 150, 200, or 250 μM in the presence of dexamethasone. **b**, **c** The streaked plate assay was used to test the migration ability of HUVECs. The medium contained CoCl_2_ at 50, 100, 150, 200, or 250 μM in the presence of dexamethasone. The dotted lines highlight the linear scratch/wound for each group of cells. **d** Cell viability at 24 or 48 h was measured by the CCK-8 assay. **e** Real-time PCR was used to detect the expression of angiogenesis-related genes in HUVECs treated with 50 μM CoCl_2_ in the presence of dexamethasone for 72 h. *p < 0.05, compared with HUVECs treated with dexamethasone. *n* = 3

Studies have shown that hypoxia can stimulate angiogenesis. To determine whether hypoxia can alleviate the inhibitory effect of dexamethasone on angiogenesis, the following experiment was performed. CoCl_2_ was used to simulate hypoxia. CoCl_2_ (50, 100, 150, 200, or 250 μM) was added to EGM to help HUVECs form capillary-like structures on Matrigel in the presence of dexamethasone. The results suggested that both 50 and 100 μM CoCl_2_ could significantly restore the capillary-like structures of HUVECs (Fig. 5a). Further research showed that the migration ability of HUVECs was improved in all hypoxia groups, especially in the groups treated with 50 and 100 μM CoCl_2_ (Fig. 5b, c). A CCK-8 assay was used to measure the proliferation capacity of HUVECs. The results suggested that HUVEC proliferation in the groups treated with 50 and 100 μM CoCl_2_ was improved significantly at 48 h (Fig. 5d). Furthermore, the q-PCR results showed that the expression of VEGF-A was significantly increased, while TIMP-3 expression was decreased (Fig. 5e).

### Hypoxia promoted HUVEC angiogenesis via HIF-1α

**Fig. 6:**
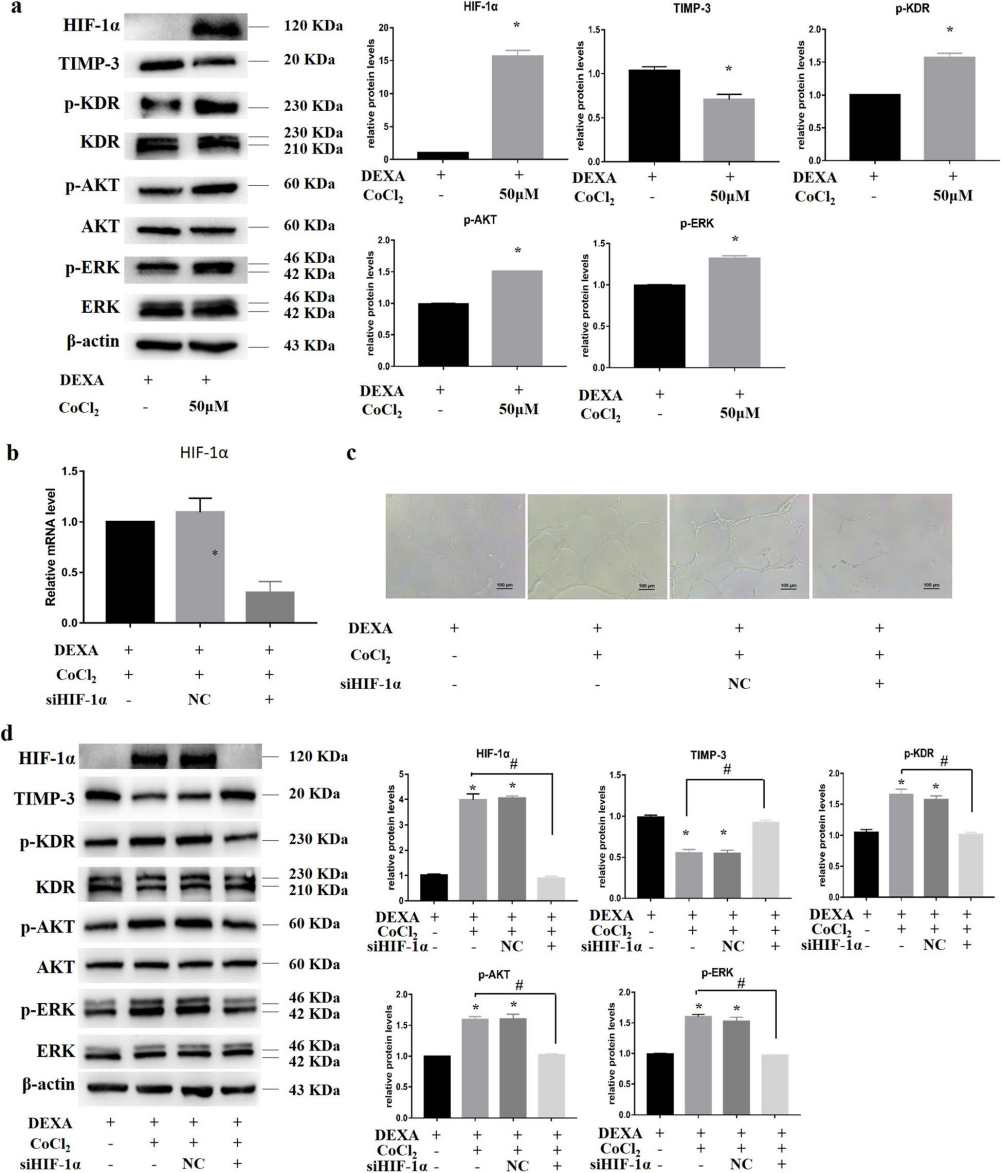
Hypoxia restored HUVEC angiogenesis via HIF-1α. **a** Western blotting was used to detect the expression of angiogenesis-related proteins in HUVECs treated with 50 μM CoCl_2_ for 72 h, and protein expression was quantified by ImageJ software. **b** HIF-1α gene expression was effectively silenced by specific siRNA sequences. **c** A Matrigel tube assay was used to detect the angiogenic capacity of HUVECs with HIF-1α silencing. **d** After HUVEC HIF-1α was silenced, the protein expression of angiogenic signaling molecules at 72 h was determined by Western blotting. *p < 0.05, compared with HUVECs treated with CoCl_2_. ^#^*p* < 0.05, compared with HUVECs treated with siNC. *n* = 3

In the following experiment, 50 μM CoCl_2_ was added to explore the mechanism by which hypoxia restores the capillary-like structures of HUVECs inhibited by dexamethasone. First, Western blotting was performed to investigate the expression of angiogenesis-related proteins under hypoxic conditions. The data showed a significant increase in the protein expression of HIF-1α, while TIMP-3 expression was decreased. In addition, VEGF-A protein expression was increased, and the phosphorylation level of KDR and its downstream targets AKT and ERK were significantly restored (Fig. 6a).

To confirm that HIF-1α plays a key role in vascular restoration under hypoxic conditions, *HIF-1α* was silenced with siRNA (Fig. 6b). Matrigel experiment results showed that hypoxia restored dexamethasone-inhibited angiogenesis, but this process was disrupted by silencing *HIF-1α* (Fig. 6c). siRNA-mediated HIF-1α gene silencing significantly decreased the protein expression of HIF-1α, while TIMP-3 expression was increased (Fig. 6d). The phosphorylation levels of KDR and its downstream factors AKT and ERK were also significantly decreased. These results indicate that hypoxia reduces TIMP-3 expression through HIF-1α, leading to increased phosphorylation of KDR and its downstream factors AKT and ERK.

### Hypoxia-preconditioned HUVECs were cocultured with MSCs to promote osteogenesis and angiogenesis

**Fig. 7:**
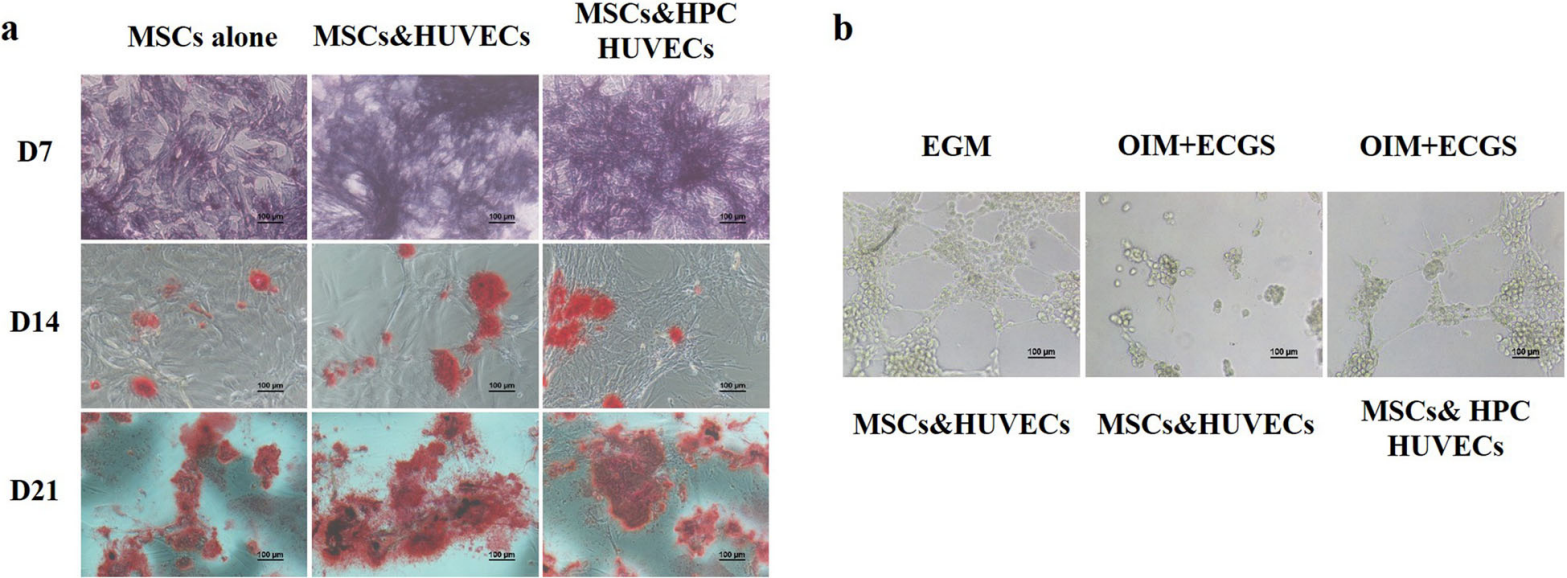
Hypoxia-preconditioned HUVECs were cocultured with MSCs to promote osteogenesis and angiogenesis. **a** MSCs were cultured in alone or were cocultured with HUVECs or with hypoxia-preconditioned HUVECs (HPC HUVECs). The OIM was changed every 3 days, NBT/BCIP staining was performed to detect ALP enzyme activity on day 7, and alizarin red staining was performed to detect calcium deposition on days 14 and 21. Scale bar, 100 μm. **b** A Matrigel tube assay was performed to test the angiogenic ability of HUVECs. Scale bar, 100 μm

We found that hypoxia-preconditioned HUVECs could promote MSCs to express ALP on day 7 and to accumulate mineralized nodules on days 14 and 21 (Fig. 7a). The Matrigel tube assay results showed that hypoxia-preconditioned HUVECs partially restored the capillary-like structures in the coculture system in the presence of OIM (Fig. 7b).

## Discussion

Bone tissue engineering with HUVEC-based prevascularization could accelerate the process of bone repair. However, studies on the MSC-HUVEC coculture system with OIM showed that HUVECs promoted MSC osteogenesis, but HUVEC angiogenesis was inhibited. Further analysis showed that dexamethasone in OIM inhibited HUVEC angiogenesis.

**Fig. 8:**
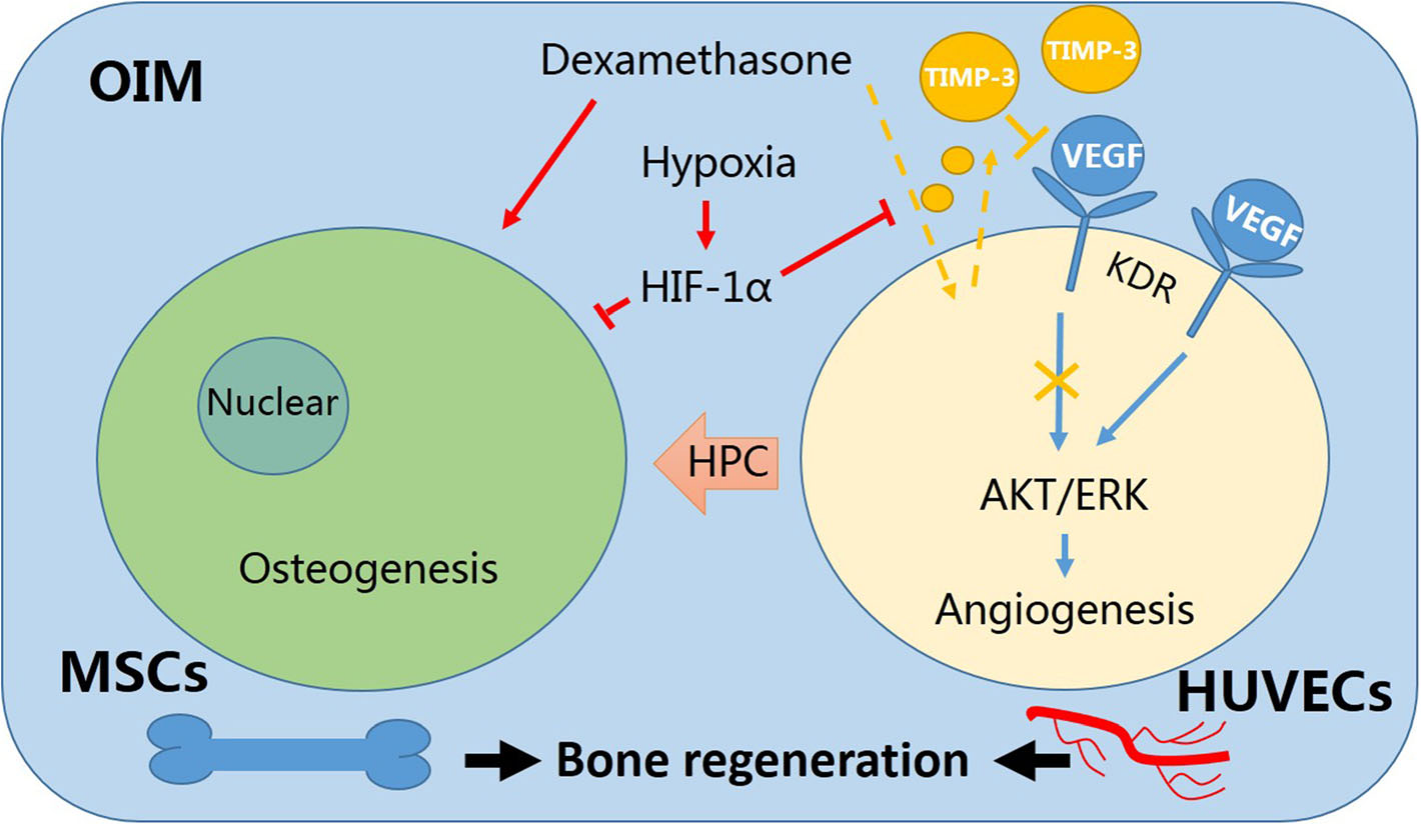
Proposed mechanism by which hypoxia alleviates the dexamethasone-induced inhibition of angiogenesis in the coculture system with OIM

A proposed mechanism of action is shown in Fig. 8. Dexamethasone, an indispensable component in OIM, promotes MSC osteogenesis. However, in this study, it was found that dexamethasone could stimulate HUVECs to secrete TIMP-3. This action prevented the phosphorylation of KDR and its downstream factors AKT and ERK. Subsequently, the proliferation and migration of HUVECs were inhibited, and HUVEC angiogenesis was limited. In addition, hypoxia was found to reduce the expression of TIMP-3 through HIF-1α. Hypoxia could alleviate dexamethasone-induced inhibition of angiogenesis in the coculture system with OIM. Moreover, hypoxia-preconditioned (HPC) HUVECs also promoted MSC osteogenesis.

VEGF-A is a key mediator of angiogenesis and mainly activates the phosphorylation of the tyrosine kinase receptor VEGFR [24]. VEGFR has three main subtype structures: VEGFR1 (Flt-1), VEGFR2 (KDR), and VEGFR3 (Flt-4). VEGFR1 and VEGFR2 mediate physiological and pathophysiological angiogenesis, while VEGFR3 mediates lymphangiogenesis. Although VEGF-A has a higher affinity for Flt-1 than KDR, KDR responds more effectively. After VEGF-A binds to KDR, the phosphorylated Y1175 residue recruits steroid receptor coactivator (Src) Homology 2 Domain Containing Adapter Protein B (SHB), activates focal adhesion kinase (FAK), and affects cell migration. The activation process of SHB also involves PI3K/AKT, which are Src substrates. AKT activates the expression of CyclinD1 via GSK-3 and mTOR to achieve cell cycle transition from G1 to S phase, thus promoting cell proliferation [25, 26]. AKT also induces cell migration via the GSK-3β/β-Catenin signaling pathway [27]. In addition, Y1175 residues recruit phospholipase C (PLC-γ), trigger Ca^2+^-dependent signaling, and activate ERK signals. ERK promotes NF-κB entry into the nucleus to regulate the expression of related genes, thus inducing cell proliferation and migration [28–30]. Similar to the results of other studies, the phosphorylation of KDR Y1175 residues was also very important for the formation of the vascular network structure in the present study. Inhibition of KDR phosphorylation reduces the proliferation and migration of HUVECs, and the vascular-like network structure will fail to form.

Dexamethasone can stimulate the expression of TIMP-3 in a variety of cells [18, 19]. Traditionally, as a potential tumor suppressor gene, TIMP-3 specifically suppresses the activity of MMPs, leading to the inhibition of tumor invasion, angiogenesis and metastasis. In clinical trials, low TIMP-3 protein levels in patients are associated with poor prognosis in nonsmall cell lung cancer patients [31]. Recent studies have shown that TIMP-3 can compete with VEGF-A for binding to KDR, leading to the inhibition of blood vessel generation [32]. In tumor tissues, overexpression of TIMP-3 significantly reduces the vascular density of tumors, resulting in a smaller tumor volume [33]. The diterpenoid lactone andrographolide, the active compound of *Andrographis paniculata*, has antitumor activity. This compound inhibits miR-21-5p, promotes the expression of TIMP-3, prevents the phosphorylation of KDR and its downstream factor MAPK, and inhibits the proliferation and migration abilities of HUVECs, blocking tumor angiogenesis [34, 35]. In this study, increased expression of TIMP-3 after treatment with dexamethasone was also observed. The increased level of TIMP-3 in HUVECs reduced the phosphorylation of KDR, AKT, and ERK, thus inhibiting cell migration and the formation of a vascular-like network.

There are some methods to remedy dexamethasone-mediated vascular depression. Dexamethasone harmed endochondral ossification by inhibiting angiogenesis in the chicken embryo model, and vascular inhibition could be reversed by the addition of insulin-like growth factor [36]. In the dexamethasone-induced rat femoral head necrosis model, vitamin K_2_ improved the survival rate, migration, and angiogenesis of EAhy926 cells [37]. In a tumor model, dexamethasone decreased the expression of VEGF in tumor cells but did not affect the expression of VEGFR in HUVECs, and this inhibition could be reversed by hypoxia [38]. Similarly, in the present study, HIF-1α induced by hypoxia reduced the expression of TIMP-3. It has also been reported that hypoxia can upregulate proangiogenic factors and downregulate antiangiogenic proteins, such as TIMP-3 and TIMP-1, also improving angiogenesis [20].

Cobalt ions are known to mimic hypoxia by artificially stabilizing the transcription factor HIF-1α. In this study, CoCl_2_ was used to alleviate vascular inhibition in the MSC-HUVEC coculture system. Previous experiments showed that 100 μM CoCl_2_ had the best recovery effect on HUVEC angiogenesis, but it was found that OIM supplemented with 100 μM CoCl_2_ inhibited MCS osteogenesis. Therefore, HUVECs were pretreated with 50 μM CoCl_2_ and then cocultured with MSCs in OIM. The effect of hypoxia on the osteogenesis of MSCs is still controversial. However, a large number of studies have shown that hypoxia or overexpression of HIF-1α in cells is beneficial for MSC osteogenic differentiation [39, 40]. However, some studies have shown that hypoxia upregulates TWIST expression through HIF-1α and inhibits the expression of RUNX2, thereby inhibiting the osteogenic differentiation of MSCs [41]. The Notch signaling pathway is upregulated under hypoxic conditions to maintain cell stemness, which does not support MSC osteogenesis [42]. Similarly, in these studies, the results showed that CoCl_2_ was not conducive to MSC osteogenesis. However, high-density oligonucleotide arrays indicated that genes induced by CoCl_2_ and hypoxia do not overlap in human hepatocellular carcinoma cells [43]. Therefore, it will be necessary to systematically evaluate the application of hypoxia in prevascularized bone tissue engineering.

## Conclusion

OIM was found to inhibit the HUVEC angiogenesis in cocultures of MSCs and HUVECs in OIM. TIMP-3 expression was stimulated by dexamethasone and was identified as an angiostatic factor that competes with VEGF-A to bind to/with KDR, thus inhibiting the angiogenesis of HUVECs. Hypoxia downregulated TIMP-3 expression via HIF-1α. The coculture system of MSCs and hypoxia-preconditioned HUVECs showed stronger osteogenesis and angiogenesis. These results revealed the critical role of TIMP-3 in angiogenesis, and a hypoxia strategy was discovered for prevascularization in bone tissue engineering.

## Data Availability

The datasets used and/or analyzed during the current study are available from the corresponding author on reasonable request.

## Abbreviations

BMSCs: Bone marrow-derived mesenchymal stem cells
HUVECs: Human umbilical vein endothelial cells
OIM: Osteogenic induction medium
DEXA: Dexamethasone
KDR (VEGFR2): Vascular endothelial growth factor receptor 2
TIMP-3: Tissue inhibitor of metalloproteinase
VEGF: Vascular endothelial growth factor
ERK: Extracellular regulated protein kinases
AKT (PKB): Protein kinase B
HIF-1α: Hypoxia-inducible factor-1α
DMEM: Dulbecco’s modified Eagle’s medium
β-gpd: β-Glycerophosphate disodium
L-Vc: L-Vitamin C
eNOS: Endothelial nitric oxide synthase
CoCl_2_: Cobalt chloride
EGM: Endothelial cell growth medium supplemented with ECGS
ALP: Alkaline phosphatase
qRT-PCR: Quantitative real-time PCR
HPC HUVECs: Hypoxia-preconditioned HUVECs
